# A comparison of peer change agent selection methods: Evidence from a high-school based suicide preventive intervention

**DOI:** 10.1186/s12889-022-13372-w

**Published:** 2022-05-16

**Authors:** Trevor A. Pickering, Peter A. Wyman, Thomas W. Valente

**Affiliations:** 1 Department of Population & Public Health Sciences, Keck School of Medicine of USC, University of Southern California, Los Angeles, CA USA; 2 Department of Psychiatry, School of Medicine and Dentistry, University of Rochester, Rochester, NY USA

**Keywords:** Peer leaders, Social networks, Diffusion of innovations, Social connectedness, School intervention, Peer messaging, Friendship networks, Social support

## Abstract

**Background:**

Peer-led interventions for adolescents are effective at accelerating behavioral change. The Sources of Strength suicide preventive program trains student peer change agents (peer leaders) in secondary schools to deliver prevention messaging and conduct activities that increase mental health coping mechanisms. The program currently has school staff select peer leaders. This study examined potential for more efficient program diffusion if peer leaders had been chosen under network-informed selection methods.

**Methods:**

Baseline assessments were collected from 5,746 students at 20 schools. Of these, 429 were selected by adults as peer leaders who delivered intervention content through the school year. We created theoretical alternate peer leader sets based on social network characteristics: opinion leadership, centrality metrics, and key players. Because these sets were theoretical, we examined the concordance of these sets with the actual adult-selected peer leaders sets and correlated this metric with diffusion of intervention modalities (i.e., presentation, media, communication, activity) after the first year.

**Results:**

The sets of adult-selected peer leaders were 13.3%—22.7% similar to theoretical sets chosen by other sociometric methods. The use of friendship network metrics produced peer leader sets that were more white and younger than the general student population; the Key Players method produced more representative peer leader sets. Peer opinion leaders were older and more white than the general population. Schools whose selected peer leaders had higher overlap with theoretical ones had greater diffusion of intervention media and peer communication.

**Conclusions:**

The use of network information in school-based peer-led interventions can help create more systematized peer leader selection processes. To reach at-risk students, delivery of an indirect message, such as through a poster or video, may be required. A hybrid approach where a combination of visible, respected opinion leaders, along with strategically-placed key players within the network, may provide the greatest potential for intervention diffusion.

## Background

Behavior change interventions, when delivered in the context of a social network (e.g., a school or workplace), can be more effective when members of the community are used to help implement the diffusion of the intervention (i.e., “peer leaders” or “peer change agents”). Peer-led network interventions are a promising approach for reducing health behavior problems among adolescents and young adults, having reduced HIV risk behaviors [1], cigarette smoking [2], and risk factors for suicidal behaviors [3]. The effectiveness of this approach stems from peer leaders/educators being seen as more credible than adults at delivering intervention messaging [4–6], being role models who persist in the community after the intervention has ended [1], and having access to informal routes of communication which can be essential to reaching less-engaged students at school [7, 8].

Schools are an ideal setting for peer-led interventions as they contain a bounded population that can provide network information, serve a broad population of youth, and provide a setting for peer socialization [9]. Still, few peer-led interventions are widely used in the school setting, and research on implementation processes and practices of peer-led programs is in its early stages [10]. One outstanding set of implementation questions concerns the selection of peer leaders in these programs: how many are required, what type of training is necessary, and how should they be selected? This study addresses how to optimize the selection of peer leaders in a school-based intervention context.

The exact demographic and sociometric characteristics of optimal peer leaders has been the subject of recent investigations. One consistent finding, congruent with network theory, is that selecting influential individuals as these change agents results in superior diffusion of information through a given network compared to randomly-selected individuals [11, 12]. Perhaps the most established method of defining “influential” individuals is opinion leadership [13], but a complement of methods are available to select respected opinion leaders in networks [14]. In a school-based intervention, the most powerful of these is to collect and use sociometric information from the entire school network. The ability to ascertain the students who are friends, leaders, admired, or respected (to name a few) can provide valuable information when making informed peer leader selection. Without this information, peer-led interventions have had to rely on methods such as self-selection [15], staff selection [16], or a combination of both [17]. Other approaches can be employed when network information is limited, such as selecting the friends of randomly selected individuals and using these friends instead as the peer change agents [18, 19]. While effective, these methods do not take advantage of a full network census.

School-based interventions allow the collection of friendship relational data at school, and when used to inform peer leader selection this information can provide a more powerful intervention compared to uninformed selection [20]. The use of a single algorithm to identify “influential” individuals, though, may ignore several facets of interpersonal influence that operate on different levels. For example, there may be strategic positions within a network that are optimal for intervention diffusion [11, 21]. Additionally, Diffusion of Innovations theory suggests that individuals who are similar to others in their network (i.e., homophilous) are more likely to spread information to peers [22], a finding that has been replicated in subsequent studies [23, 24]. In addition to opinion leadership, it is clear that network position and representativeness should be considered when selecting peer leaders.

To explore the ways in which selection methods may influence whom is chosen as peer leaders, the current study examines the sociometric and demographic characteristics of peer leader sets produced through several different theoretical selection methods. For each of the peer leader sets we examine: 1) sociometric characteristics, 2) the distance of the peer leader set to at-risk students (individuals with suicidal thoughts or behaviors, individuals peripheral in the network, individuals isolated from adults) who are not expected to be reached as well by traditional interventions, 3) the extent of clustering of peer leaders within each set, and 4) the representativeness of these peer leaders based on demographic characteristics. We additionally examine the concordance of empirical adult-selected peer leaders with these theoretical peer leader sets to see if concordance relates to message diffusion observed in the intervention. We hypothesize that in schools where the current adult-selected peer leader sets have higher concordance with theoretical sociometric ones, student exposure to intervention will be higher across the four measured exposure modalities.

## Methods

### Schools and student enrollment

Data for this study comes from a type I hybrid effectiveness-implementation trial of a peer-led suicide prevention program, Sources of Strength [3], in 40 high schools. Schools were in predominantly rural, small town, and micropolitan communities of New York (*n* = 31) and North Dakota (*n* = 9), based on Rural Urban Commuting Area scores. Schools were selected for enrollment in Sources of Strength based on location in a county or public health region with past five-year youth suicide rates above the state average (24.40 and 5.19 per 100,000 in North Dakota and New York, respectively, for youth 15–19 in 2009–2011). The 40 high schools were enrolled in four cohorts (2010–2013), with schools stratified by size and location; matched pairs were subsequently randomized into either immediate implementation or wait-list conditions. The 20 high schools randomized to begin immediate implementation of Sources of Strength are included in this study (16 in New York, 4 in North Dakota). The schools ranged in student population size from 63–1,207 students (M = 366). Two schools served Native American reservations. All students in grades 9 – 12 were invited for repeated longitudinal assessments to evaluate program diffusion and impact [25]. The University of Rochester IRB approved the study protocol.

### Peer leader selection and role

Peer leader selection was preceded by baseline assessment of the school’s student population and training of adult staff in each school (i.e., adult advisors), whose role included recruiting student peer leaders and facilitating their role as prevention agents. Identical, standardized procedures were used in each school to recruit peer leaders. This process consisted of distributing nomination forms to staff members which asked for nominations of up to 6 students whose “voices are heard” by other students. Nominations were reviewed to select a target of 5–10% of students who reflected diverse population groups within their school. The size of the peer leader team varied by high school, contingent on school size and staff selection. A total of 959 students were invited (19–86 per school), with 789 (83.2%) enrolling with parent permission and youth assent. Of these, 459 (9–45 per school) were retained as active peer leaders through the end of the first school year. Selected peer leaders and their adult advisors participated in a 5-h training covering natural coping resources (e.g., trusted adults, family support, positive activities) and their role in school-wide dissemination of those strengths. Following training, peer leaders were invited to participate in bi-weekly meetings to plan and carryout prevention campaigns to spread ‘sources of strength’ and normalize engaging adult support for students in crisis or suicidal.

### Survey Variables

#### Demographics

The baseline survey administered to all students collected information on student sex, ethnicity (white vs. nonwhite), and grade level.

#### Suicidal thoughts and behaviors

Using questions from the Youth Risk Behavior Survey [26], each student was asked whether in the preceding 12 months he/she had: seriously considered suicide; planned suicide; made one or more suicide attempts; or made an attempt that resulted in injury requiring medical treatment. Three categories were created to describe suicidal behavior: suicide attempt with or without injury, seriously considered suicide without attempt, and no suicidal thoughts or behaviors.

#### Intervention diffusion

Diffusion of the Sources of Strength intervention was measured at the end of the first year and categorized into four different dichotomous modalities corresponding to various levels of engagement which included awareness of, communication about, and active participation in the intervention [27]. Students were asked about these exposures, preceded by the phrase, “Some students in your school have been trained as Peer Leaders in a program called Sources of Strength.” Students were subsequently asked about:

1. *Presentation or assembly* attendance consisted of answering “yes” to either: Have you seen a presentation or assembly about… (a) strengths that help teens get through hard times?, or (b) helping suicidal teens by getting adults involved? Example presentations included peer leaders leading presentations in their class about the “Sources of Strength wheel” and a source they felt they were strong in.
2. *Poster or video viewing* was assessed by answering “yes” to: Have you seen posters or videos at school about strengths? Example posters included pictures of the eight different sources of strength.
3. *Direct peer communication participation* was based on answering “yes” to either: Has a friend or other student… (a) told you about Sources of Strength?, or (b) talked to you about using strengths?
4. *Intervention activity participation* consisted of answering “yes” to either: (a) Have you participated in a Sources of Strength activity such as adding your trusted adult to a poster?, or (b) Has a friend or other student asked you to name adults you can go to for help?

### Analysis

#### Theoretical peer leader selection

**Fig. 1:**
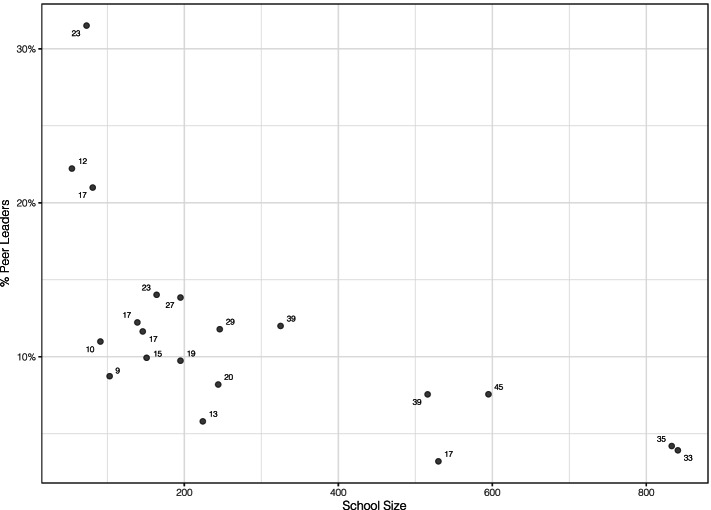
Percent of students selected as peer leaders who participated through the full school year, by school size. Points are labeled as number of peer leaders in the given school

The number of adult-selected peer leaders (APL) varied by school (Fig. 1). For a given school *i* with a set of *n* APL, a theoretical set of *n*_*i*_ peer leaders were identified by each of the following methods. Whenever a ranked method produced a tie, students were randomly selected to break the tie.

1. *Peer Opinion Leaders (POL).* Students were asked to name up to three students in school who they considered to be “student leaders who others listen to.” Nominations were summed to produce the total nominations received per student (opinion leader in-degree). The top *n*_*i*_ opinion leaders at each school were selected as POL.
2. *Friendship Network Opinion Leaders (FNOL)*. Students were asked to name up to seven students in school who are their closest friends. These nominations produced several individual-level network variables, including: (a) In-degree (*FNOL-In)*: the number of friendship nominations received from others; (b) Coreness *(FNOL-Co)*: for each student, the k-core is the maximal subgraph in which each vertex has degree *k*, with larger values indicating membership in a more cohesive, interconnected group of friends; (c) Closeness *(FNOL-Cl)*: the reciprocal sum of distances to each other student in the network, indicating central proximity to all other students; and (d) Betweenness *(FNOL-Bt)*: the number of times an individual is in the shortest path connecting all other nodes, an indicator of potential to bridge disparate groups. The iGraph package in R [28] was used to compute all individual-level friendship network variables. For each metric, the top *n*_*i*_ students were selected as FNOL.
3. *Key Players (KPL)*. The key players algorithm identifies key players for the purpose of optimally diffusing information through a network [29]. Borgatti notes one practical implementation of this algorithm is to select a small set of a population as seeds to diffuse practices or attitudes that promote health. The approach selects a set of maximally connected individuals who tend to be equally spaced throughout the network. The approach addresses the “redundancy problem,” the tendency of highly central nodes to be structurally equivalent and therefore connected to the same individuals. The key players algorithm (KPP-POS) was performed using the InfluenceR package in R [30] to identify *n*_*i*_ KPL in each school.
4. *Hybrid Methods (HPL).* Three hybrid methods of peer leader identification were implemented. In each case, representative samples of the population were taken by stratifying the school population by ethnicity, sex, and grade level and choosing a proportional number of peer leaders within each stratum, rounded down. This method produced *n-k* total peer leaders per school. Then, the key players algorithm was used to select *k* remaining peer leaders within that school. The peer leader sets chosen under the hybrid approach were selected by the following algorithms: (a) Influence-weighted *(HPL-Inf)*: the students with the highest opinion leader in-degree and friendship in-degree were chosen within each stratum. [2] Centrally-weighted *(HPL-Cen)*: the students with the highest closeness and betweenness were chosen within each stratum. [3] Structurally-weighted *(HPL-Str)*: the students were chosen as with the influence-weighted methods, but restricted to no more than 2 per stratum. This produced a greater proportion of peer leaders being chosen through the key players algorithm.

#### Assessment metrics

Theoretical peer leader sets were evaluated by assessing sociometric and demographic characteristics, which were standardized within school to produce z-scores. These scores were averaged across all peer leaders to produce a mean value with respect to the general student body at each school (e.g., a value of 1 would indicate one standard deviation difference in that metric compared to the average for all students). To account for within-school clustering, reported means and standard errors are derived from mixed-effect models that included only a random intercept for school.

**Fig. 2:**
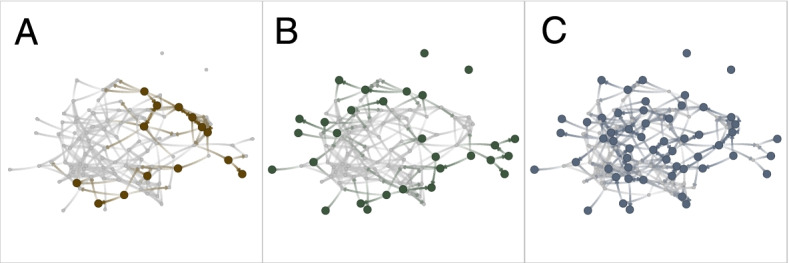
Distribution of “at-risk” students in one sample school: students with suicide ideation or attempt **A**, students in the network periphery **B**, and students who did not name a trusted adult **C**

1. *Selection Concordance.* To address the concordance of the APL with the proposed theoretical ones, we measured the percent of students in the theoretical peer leader sets who were also in the APL set. We additionally computed concordance among all other theoretical peer leader selection methods. For example, if the school-level concordance between APL and POL methods was 20%, then this indicates 20% of the peer leaders selected based on opinion leadership at that school had also been chosen as adult-selected peer leaders.
2. *Sociometric Characteristics.* The average in-degree, out-degree, coreness, closeness, betweenness, and opinion leader nominations were computed for each individual and standardized within school.
3. *Clustering.* To determine the extent of peer leader clustering, for each selection method we calculated the average number of peer leaders within one step of (i.e., directly connected to) any given peer leader, based on the friendship network.
4. *Representativeness.* To assess demographic representation, for each selection method we calculated the sex, race, age, and grade level of peer leaders and compared these values to the school mean.
5. *Reach.* To determine the proximity of selected peer leaders to at-risk students, we calculated the distance of each peer leader to the closest student in each of the three risk categories. A lower value reflected being closer in friendship steps to these peers. Risk categories included: 1) indicating suicide ideation or suicide attempt, 2) being in the periphery of the network [31], and 3) naming no trusted adults at school. In the case that a peer leader was disconnected from all other students within a risk category, the maximum distance in the network was assigned. One school had no suicide attempts and was excluded from statistics on distance to closest student with attempt. Figure 2 shows the distribution of at-risk students within the network of one sample school. The smallest risk group was suicidality (school-level proportion = 15.4%), followed by peripheral students (16.2%), with a considerable number of students not naming a trusted adult (32.0%).

Data import, cleaning, and analysis were performed in R v4.1.1 [32]. The creation of network objects and network metrics was performed with the iGraph package. To determine the relationship between concordance of peer leader selection methods with intervention diffusion, schoolwide percent exposure to the four Sources of Strength modalities was regressed against the percent concordance with each peer leader selection method, with and without adjusting for log-transformed school size. Regression analyses on these 20 school-level observations was performed in R using the glm package.

## Results

### Sample

Across the 20 schools, average enrollment in the evaluation was 82.2% (range 65.9–98.3%) for a final sample of 5,746 students (range 54–841 per school). Of these, 4,026 participants completed information on exposure to the intervention Sources of Strength at the end of the first year. Demographic characteristics of all students participating in the baseline survey and survey at the end of the first school year are presented in Table 1.

**Table 1:** Demographic characteristics of students participating in the Sources of Strength assessments (*n* = 5,746)

Variable	School-Level Mean (SD)	School-Level Range
School Size ^a^	287 (244)	54—841
Sex—Male	51.1% (3.44%)	44.5%—59.2%
Race—White	80.4% (22.8%)	1.02%—98.9%
Age	15.7 (0.19)	15.5—16.2
Suicide Ideation	6.6% (2.1%)	2.9%—12.0%
Suicide Attempt	6.6% (3.0%)	0%—13.9%

^a^# with baseline survey included in social network analyses

### Selection concordance

The students chosen by theoretical selection methods generally had low correspondence to the students empirically chosen by adults (Table 2). The amount of concordance with APL was as low as 13.3% for KPL and as high as 21.6% for POL. Among all theoretical selection methods, concordance was the highest between FNOL-Cl and FNOL-Bt (54.2%) and lowest between FNOL-Co and FNOL-Bt (11.1%). FNOL-Dg had consistently high concordance, as it was related to POL (35.5%), FNOL-Co (31.8%), FNOL-Cl (32.9%), FNOL-Bt (30.9%), and even to KPL (30.3%).

**Table 2:** Concordance among peer leader selection methods. For each pair of peer leader sets, displayed are the number (and %) of students who appear in both sets

	APL	POL	FNOL-Dg	FNOL-Co	FNOL-Cl	FNOL-Bt	KPL
APL	459 (100%)						
POL	99 (21.6%)	459 (100%)					
FNOL-Dg	85 (18.5%)	163 (35.5%)	459 (100%)				
FNOL-Co	70 (15.3%)	94 (20.5%)	146 (31.8%)	459 (100%)			
FNOL-Cl	71 (15.5%)	93 (20.3%)	151 (32.9%)	84 (18.3%)	459 (100%)		
FNOL-Bt	66 (14.4%)	88 (19.2%)	142 (30.9%)	51 (11.1%)	249 (54.2%)	459 (100%)	
KPL	61 (13.3%)	86 (18.7%)	139 (30.3%)	57 (12.4%)	70 (15.3%)	110 (24%)	459 (100%)
HPL-Inf	104 (22.7%)	258 (56.2%)	227 (49.4%)	88 (19.2%)	128 (27.9%)	142 (30.9%)	127 (27.7%)
HPL-Cen	102 (22.2%)	205 (44.7%)	246 (53.6%)	94 (20.4%)	158 (34.4%)	185 (40.3%)	136 (29.6%)
HPL-Str	98 (21.3%)	217 (47.2%)	203 (44.2%)	78 (17.0%)	103 (22.4%)	127 (27.7%)	153 (33.3%)

### Sociometric characteristics of peer leader sets

Each theoretical sociometric method produced individuals with the highest values of the respective sociometric characteristic (Table 3). The set of APL also had higher sociometric characteristics than the average student, but these values were lower than other network-informed selection methods. Consistent with their role as respected members of the community, POL had high standardized values of in-degree (M =  + 1.13, SE = 0.06). KPL had higher values of each sociometric compared to the general school population, but these values were modest in relation to peer leaders chosen through other sociometric selection methods.

**Table 3:** Metrics of the overall sample and 459 peer leaders chosen under various methods. The column of means and SD reflect values from all students in the study. For all other columns, displayed is the average score of peer leaders for each selection method in relation to their school-level mean value. Peer leader difference scores are presented in z-score units (number of standard deviations difference from the overall student population value). Bold values differ significantly from 0 at *p* < .05

Metric			M (SD)	**Selection Method**						
				APL	POL	FNOL-Dg	FNOL-Co	FNOL-Cl	FNOL-Bt	KPL
*Social Network Metrics*										
In-Degree		4.08 (2.94)		** + 0.40** **(0.06)**	** + 1.13** **(0.06)**	** + 2.00** **(0.09)**	** + 0.93** **(0.06)**	** + 1.02** **(0.07)**	** + 0.97** **(0.07)**	** + 0.79** **(0.13)**
Out-Degree		4.79 (2.69)		** + 0.22** **(0.06)**	** + 0.34** **(0.04)**	** + 0.50** **(0.04)**	** + 0.56** **(0.04)**	** + 0.67** **(0.04)**	** + 0.68** **(0.03)**	** + 0.45** **(0.06)**
Coreness		4.97 (1.96)		** + 0.34** **(0.08)**	** + 0.65** **(0.03)**	** + 0.85** **(0.03)**	** + 1.17** **(0.06)**	** + 0.71** **(0.05)**	** + 0.54** **(0.03)**	** + 0.33** **(0.06)**
Closeness		0.10 (0.04)		** + 0.30** **(0.05)**	** + 0.48** **(0.05)**	** + 0.65** **(0.03)**	** + 0.60** **(0.04)**	** + 1.03** **(0.03)**	** + 0.89** **(0.03)**	** + 0.33** **(0.05)**
Betweenness		1573 (2467)		** + 0.28** **(0.05)**	** + 0.52** **(0.07)**	** + 1.04** **(0.07)**	** + 0.20** **(0.05)**	** + 1.70** **(0.09)**	** + 2.29** **(0.12)**	** + 0.28** **(0.05)**
*Connectedness*										
PLs within 1 Step				1.34 (0.06)	2.17 (0.08)	2.43 (0.08)	3.66 (0.11)	3.53 (0.09)	2.27 (0.07)	0.42 (0.04)
% Disconnected		2.9% (16.9%)		0.9% (0.4%)	-	-	-	-	-	-
*Demographics*										
Sex (% Female)		49.4% (50.0%)		** + 0.22** **(0.05)**	+ 0.08 (0.05)	-0.04 (0.05)	+ 0.10 (0.07)	+ 0.11 (0.07)	+ 0.04 (0.05)	-0.02 (0.05)
Race (White)		72.1% (44.9%)		+ 0.03 (0.05)	** + 0.15** **(0.05)**	** + 0.11** **(0.05)**	+ 0.07 (0.06)	** + 0.12** **(0.04)**	+ 0.00 (0.05)	-0.07 (0.05)
Age		15.7 (1.3)		**-0.14** **(0.06)**	** + 0.35** **(0.06)**	-0.10 (0.06)	-0.12 (0.12)	**-0.26** **(0.07)**	**-0.17** **(0.04)**	+ 0.00 (0.05)
Grade Level		10.4 (1.1)		-0.03 (0.05)	** + 0.47** **(0.05)**	-0.03 (0.05)	-0.06 (0.13)	**-0.20** **(0.07)**	**-0.17** **(0.04)**	-0.03 (0.05)
Suicide Ideation		8.8% (28.4%)		+ 0.04 (0.05)	-0.08 (0.05)	**-0.15** **(0.03)**	-0.08 (0.05)	-0.07 (0.04)	0.02 (0.05)	-0.04 (0.04)
Suicide Attempt		7.6% (26.5%)		-0.04 (0.05)	**-0.10** (0.04)	-0.01 (0.05)	-0.09 (0.05)	**-0.14** **(0.04)**	-0.04 (0.05)	0.04 (0.05)
*Average distance*										
…to closest SI	2.07 (1.81)			**-0.13** **(0.04)**	**-0.16** **(0.04)**	**-0.29** **(0.04)**	**-0.22** **(0.07)**	**-0.26** **(0.03)**	**-0.30** **(0.03)**	**-0.24** **(0.05)**
…to closest SA	2.29 (1.82)			-0.08 (0.05)	**-0.18** **(0.04)**	**-0.25** **(0.05)**	-0.09 (0.07)	**-0.18** **(0.06**)	**-0.32** **(0.03)**	**-0.28** **(0.03)**
…to closest peripheral	2.41 (1.71)			-0.08 (0.04)	**-0.20** **(0.04)**	**-0.24** **(0.03)**	-0.02 (0.06)	**-0.10** **(0.04)**	**-0.28** **(0.03)**	**-0.32** **(0.04)**
…to closest adult isolate	1.54 (1.75)			**-0.07** **(0.04)**	**-0.18** **(0.03)**	**-0.31** **(0.03)**	**-0.18** **(0.03)**	**-0.23** **(0.02)**	**-0.28** **(0.02)**	**-0.24** **(0.03)**

### Clustering within peer leader sets

**Fig. 3:**
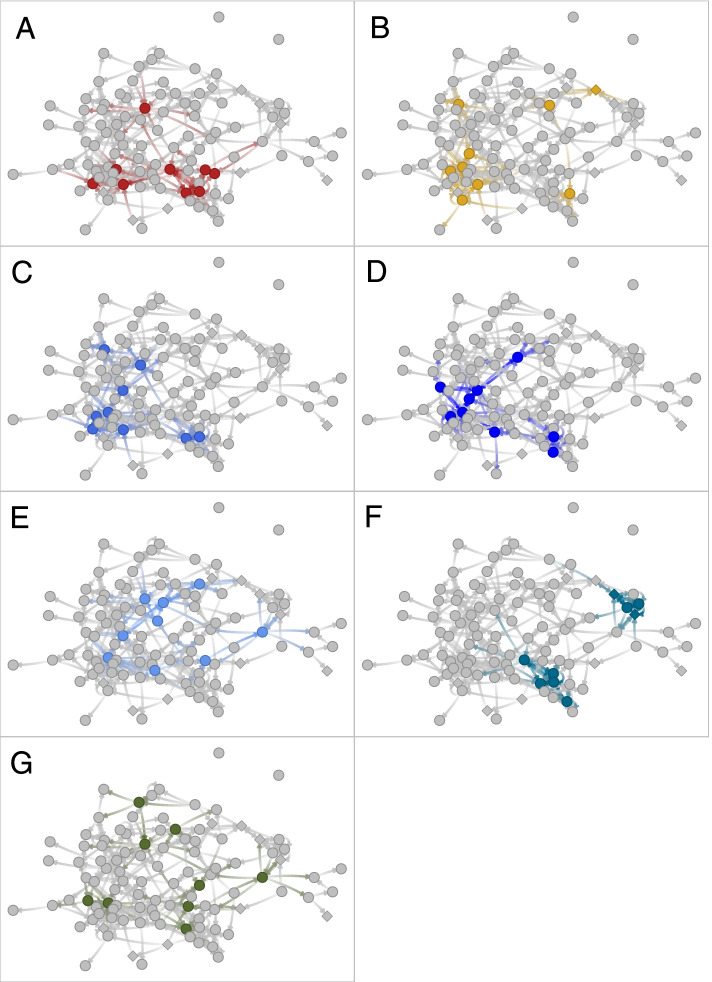
Peer leaders selected using various methods in a sample school. Methods include: APL **A**, POL **B**, FNOL-Dg **C**, FNOL-Cl **D**, FNOL-Bt **E**, FNOL-Co **F**, and KPL **G**. Students are shown as circles, except those with suicide ideation/attempt who are shown as a diamond

The largest clustering among peer leaders occurred for the FNOL-Co and FNOL-Cl; these sets of students typically had over 3 peer leaders within one friendship step (3.66 and 3.53, respectively). KPL had the fewest direct connections to other peer leaders (0.42 peer leaders within one step). While instructed to select students from diverse groups within the school, APL on average had ties to 1.34 other peer leaders. Figure 3 illustrates the general trends of clustering and network position in a sample school. Consistent with the findings in Table 3, FNOL-Co and FNOL-Cl appear highly clustered, while KPL appear to be uniformly spread through the network. POL were generally more dispersed through the network, but still tended to cluster in local pockets.

### Demographic characteristics of peer leader sets

There were large demographic differences among the sets of peer leaders produced by different methods. A greater proportion of APL were female compared to the general student population (M =  + 0.22, SE = 0.05). While APL generally matched the ethnic composition of the student populations, POL (M =  + 0.15, SE = 0.05), FNOL-Dg (M =  + 0.11, SE = 0.05), and FNOL-Cl (M =  + 0.12, SE = 0.04) produced peer leaders that were more ethnically white. APL were younger than the general student population (M = -0.14, SE = 0.06), while POL tended to be older and in a higher grade compared to other students (M =  + 0.35 & + 0.47, respectively).

### Distance to at-risk students

The proportion of peer leaders with suicide ideation and suicide attempt matched that of the general population under almost all selection methods. However, FNOL-Dg had a lower proportion with suicide ideation than the general population (M = -0.15, SE = 0.03), while POL and FNOL-Cl had a lower proportion with suicide attempt (M = -0.10 & -0.14, respectively). Every selection method produced peer leaders who were closer to at-risk students than the general population. APL and FNOL-Co, though, were not closer to students with suicide attempt or peripheral students.

### Relationship between concordance and diffusion

School-level percent concordance of APL with theoretical selection methods (i.e., “selection concordance”) was related to diffusion for some modalities (Table 4). Selection concordance was not a significant predictor of schoolwide diffusion as evidenced by attendance at a presentation, nor did it significantly predict schoolwide activity participation in analyses adjusted for school size. Schoolwide rates of direct peer communication were significantly larger when schools had peer leader sets that more closely aligned with POL, FNOL-Cl, and all HPL. In analyses adjusted for school size (ln), this effect remained significant for POL and marginal for all HPL. The largest adjusted effect was for POL concordance; a 1% increase in POL concordance was associated with a 0.82% increase in students with direct peer communication (*p* < 0.001). Having viewed a poster/video was significant for concordance with all methods except FNOL-Co. In adjusted analyses among those with suicide ideation or attempt, POL and all HPL concordance were significantly associated with having viewed intervention media.

**Table 4:** Relationship between selection method concordance and exposure to Sources of Strength across four modalities in 20 schools. Displayed are the regression coefficients that reflect the change in percent exposure associated with the change in percent concordance (SE)

*Outcome: School-level percent of all students exposed*								
	Unadjusted				School Size (ln) Adjusted			
	Presentation	Poster/Video	Direct Peer	Activity	Presentation	Poster/Video	Direct Peer	Activity
POL	0.32 (0.34)	1.05 (0.30)*	0.89 (0.22)**	-0.01 (0.39)	0.18 (0.38)	0.74 (0.29)*	0.82 (0.24)*	-0.49 (0.35)
FNOL-Dg	0.50 (0.31)	0.82 (0.31)*	0.41 (0.27)	0.10 (0.37)	0.41 (0.37)	0.41 (0.34)	0.21 (0.32)	-0.51 (0.36)
FNOL-Co	0.23 (0.37)	0.73 (0.38)	0.41 (0.31)	0.23 (0.42)	0.04 (0.41)	0.28 (0.37)	0.20 (0.34)	-0.26 (0.40)
FNOL-Cl	0.56 (0.3)	1.04 (0.27)*	0.58 (0.25)*	0.44 (0.35)	0.52 (0.37)	0.72 (0.31)*	0.46 (0.31)	-0.07 (0.38)
FNOL-Bt	0.29 (0.27)	0.83 (0.24)*	0.40 (0.22)	0.63 (0.27)*	0.11 (0.44)	0.48 (0.38)	0.23 (0.36)	0.18 (0.43)
KPL	0.46 (0.35)	0.87 (0.36)*	0.59 (0.29)	0.30 (0.41)	0.34 (0.39)	0.50 (0.34)	0.43 (0.32)	-0.12 (0.39)
HPL-Inf	0.30 (0.33)	0.89 (0.27)*	0.61 (0.22)*	0.06 (0.34)	0.19 (0.33)	0.62 (0.26)*	0.51 (0.24) +	-0.32 (0.31)
HPL-Cen	0.38 (0.33)	1.02 (0.30)*	0.66 (0.26)*	-0.03 (0.39)	0.28 (0.35)	0.76 (0.27)*	0.55 (0.27) +	-0.36 (0.34)
HPL-Str	0.55 (0.31)	1.07 (0.28)*	0.68 (0.24)*	0.23 (0.37)	0.49 (0.39)	0.74 (0.32)*	0.59 (0.30) +	-0.42 (0.38)
*Outcome: School-level percent of students with suicide ideation/attempt exposed*								
	Unadjusted				School Size (ln) Adjusted			
	Presentation	Poster/Video	Direct Peer	Activity	Presentation	Poster/Video	Direct Peer	Activity
Opinion Leader	0.44 (0.39)	1.13 (0.4)*	0.52 (0.25)*	-0.08 (0.45)	0.30 (0.44)	0.95 (0.44)*	0.56 (0.28)	-0.58 (0.42)
Degree	0.51 (0.36)	0.83 (0.41)	0.16 (0.26)	0.22 (0.42)	0.39 (0.44)	0.57 (0.49)	0.12 (0.31)	-0.35 (0.44)
Coreness	0.20 (0.43)	1.04 (0.46)*	0.06 (0.3)	0.38 (0.47)	-0.03 (0.48)	0.80 (0.51)	-0.02 (0.34)	-0.08 (0.48)
Closeness	0.41 (0.37)	0.78 (0.41)	0.27 (0.25)	0.01 (0.42)	0.24 (0.45)	0.49 (0.50)	0.28 (0.31)	-0.73 (0.42)
Betweenness	0.21 (0.32)	0.47 (0.36)	0.07 (0.22)	0.37 (0.34)	-0.21 (0.51)	-0.12 (0.58)	-0.07 (0.36)	-0.53 (0.49)
Key Players	0.30 (0.43)	0.94 (0.46)	0.43 (0.27)	0.25 (0.47)	0.12 (0.47)	0.69 (0.50)	0.44 (0.31)	-0.19 (0.46)
HPL1	0.41 (0.33)	1.08 (0.31)*	0.42 (0.21)	0.05 (0.37)	0.30 (0.36)	0.95 (0.34)*	0.43 (0.23)	-0.28 (0.35)
HPL2	0.31 (0.31)	1.01 (0.29)*	0.37 (0.20)	0.05 (0.35)	0.19 (0.34)	0.89 (0.32)*	0.39 (0.22)	-0.32 (0.34)
HPL3	0.48 (0.32)	1.11 (0.31)*	0.43 (0.21)	0.11 (0.37)	0.38 (0.36)	0.99 (0.33)*	0.45 (0.23)	-0.23 (0.36)

^*^p < .05, + *p* < .10

## Discussion

Our findings confirm that the use of network information to inform peer leader selection has promise in improving the diversity and network position of peer leader sets and potentially enhancing intervention diffusion in participating schools. We see that the intent of APL sets—namely, to contain a diverse sample of students from across the network—appears to be somewhat achieved in the Sources of Strength intervention. The current APLs tended to cluster less than those from other theoretical selection methods and had higher values of network centrality characteristics compared to the general student population. This suggests that adults may be tapping into implicitly observed information about the school network even without using formal analytic methods. Nonetheless, there is still potential to optimize peer leader selection as APL tended to be less central, more female, and less close to at-risk students compared to the other selection methods.

### The power of key players

Though the Key Players algorithm was designed to produce a set of individuals maximally connected to others in the network, it additionally performed quite well at producing a representative sample of individuals. Each other sociometric method produced peer leaders with characteristics incongruent with selection goals (e.g., FNOL selected more white, younger, female peer leaders, and POL selected more white, older peer leaders). KPL, though, aligned with the student population on all demographic characteristics, perhaps selecting individuals from various demographic clusters in the network. This finding has been shown in other research; for example, it has been suggested that the selection of Key Players be used to supplement formal leaders in order to reflect a more diverse set of group interests [33]. FNOL-Bt also contained individuals who ethnically similar to the overall student population, likely because individuals with high betweenness tend to bridge disparate groups and, in these secondary schools, groups tend to be defined by sex and race.

### Connecting to at-risk students

Prevalence of suicide ideation and attempt generally did not differ for any peer leader sets, with some exceptions. FNOL-Dg had a lower rate of suicide ideation, and one interpretation could relate to the constraints placed on popular individuals within networks. That is, popular students may have the ability to spread information and set trends within networks, but their behaviors and attitudes generally tend to be reflective of the network overall [34]. It has been shown that students in Sources of Strength schools who have suicide ideation or attempt tend to be less popular than those without suicidality on average, suicidal students are 86% as popular as non-suicidal students [25]. While popular students may have the ability to be behavioral role models, they may also be less connected and empathetic to the needs of suicidal students within the network. Indeed, we found a relationship between concordance of POLs and diffusion of direct peer communication, but this relationship disappeared in the subsample of students with suicidal thoughts and behaviors. While it is discouraging that there does not appear to be a relationship between concordance of any selection method and direct communication in the subsample of students with suicidal thoughts and behaviors in adjusted analyses, there was a significant relationship between concordance and poster/video exposure for POL and HPL methods in this subsample. Interventions attempting to reach students in this subsample may need to rely on the power of peer leaders as indirect community role models, and less on direct routes of communication.

Interventions may need to alter their peer leader selection method according to characteristics of their target population. Choosing peer leaders that are close to the population of interest (i.e., fewer steps away in the friendship network) is critical; individuals are less likely to be exposed the further they are from peer leaders, and this effect tapers off at a distance of 3 friendship steps [27]. APL were close to at-risk students, but nearly all network-informed peer leader sets contained individuals who were closer, with FNOL-Dg, FNOL-Bt, and KPL being the closest. The effects are such that if theoretical peer leader sets had been used instead of APL, on average 1 additional at-risk student could have been reached for every 2 KPL or for every 4 POL. When considering the ability to reach at-risk students within the network, network-informed selection appears superior to methods that do not use this information.

### Selection methods and diffusion

Concordance between APL and all selection methods was not related to the proportion of students who had seen a presentation. This modality involving structured peer-led campaigns was hypothesized to be the least affected by peer interactions; it therefore is not surprising that there is a diminished role of peer leaders at facilitating exposure to this modality, and thus the peer leader selection method may be less important. On the other hand, schools with high concordance between APL and POL had increased natural peer communications about Sources of Strength, which speaks to the power of POL at delivering messages. Consistent with early findings on opinion leadership [13], network position alone may not be enough to relay messages; diffusion of peer-led interventions is moderated by the recipients’ perception of opinion leadership from the messenger [35]. Additionally, simulation studies have confirmed that, beyond their position within the social network, opinion leaders have a powerful effect on product adoption because of their respected role within networks [36].

Several concordance measures were related to having seen a poster/video. This is unexpected considering our hypothesis that peer-to-peer communication would respond more to peer leader selection compared to poster/video exposure. One explanation for this could be that peer leaders affect the viewing of a poster/video not simply because of their network position, but because certain characteristics of peer leaders (e.g., charisma, enthusiasm, recognizability, relatability) may help them better deliver multimedia-based formats like posters and videos. The data indicate that POL, FNOL-Cl, and HPL may be better at influencing this modality. FNOL-Cl tend to be close to many others in the friendship network, perhaps making their messaging efforts more salient to students. Since POL tend to be older and more respected, students may be more willing to share their intervention messaging in electronic formats rather than have a direct conversation about intervention topics.

### Limitations

This study draws strength from a large data set collected across several schools. While these data come from a larger randomized control trial, the theoretical peer leader sets in this study were not implemented in schools. Future work could implement these selection methods and observe actual rates of intervention diffusion within schools. Although APL were selected according to a standardized protocol, there was some degree of subjectivity involved such that APL sets may be unreproducible. Therefore, results that pertain to concordance with APL may not be generalizable to other studies. There may be several other considerations for peer leaders that may influence diffusion that are not measured here: willingness to participate, attitudes toward the intervention, school attendance, student personality type, persuasiveness, etc. Though these characteristics may affect diffusion, they are not measured with the current survey and may not be feasible to obtain through survey methods at the beginning of a school year.

## Conclusions

The use of network information to obtain influential, representative sets of peer leaders can help create more systematized peer leader selection processes. The current adult-selected method produced peer leaders in suboptimal network positions, but network-informed methods come with challenges as well. Key players were demographically representative and close to at-risk students but may be limited in their reach. Respected opinion leaders, while older and less ethnically representative, may be better equipped to deliver indirect intervention messaging. A hybrid approach where a combination of visible, respected opinion leaders, along with key players strategically placed within the network, may provide the greatest potential for intervention diffusion. Future work should follow interventions that use these selection methods to determine how they directly translate to diffusion of interventions through the school network.

## Data Availability

Due to the study involving minors and with questions relating to sensitive topics, the datasets analyzed during this study are not publicly available, but may be available from Peter A. Wyman on reasonable request.

## Abbreviations

APL: Adult-selected peer leaders
POL: Peer opinion leaders
FNOL-In: Friendship network opinion leaders chosen by in-degree
FNOL-Co: Friendship network opinion leaders chosen by coreness
FNOL-Cl: Friendship network opinion leaders chosen by closeness
FNOL-Bt: Friendship network opinion leaders chosen by betweenness
KPL: Key players opinion leaders, HPL
Inf: Influence-weighted hybrid peer leaders
HPL-Cen: Centrally-weighted hybrid peer leaders
HPL-Str: Structurally-weighted hybrid peer leaders
